# Preoperative anemia and long-term survival in patients undergoing colorectal cancer surgery: a retrospective cohort study

**DOI:** 10.1186/s12957-023-03005-w

**Published:** 2023-04-04

**Authors:** Yixu Deng, Meilin Weng, Jun zhang

**Affiliations:** 1 Department of Anesthesiology, Fudan University Shanghai Cancer Center, No. 270 Dongan Road, Xuhui District, Shanghai, 200032 People’s Republic of China; 2 Department of Oncology, Shanghai Medical College, Fudan University, Shanghai, 200032 People’s Republic of China; 3 Department of Anesthesiology, Zhongshan Hospital, Fudan University, Shanghai, 200032 People’s Republic of China

**Keywords:** Anemia, Colorectal cancer, Surgery, Overall survival, Disease-free survival

## Abstract

**Background:**

The impact of preoperative anemia on a survival outcome and the importance of correcting preoperative anemia in patients with colorectal cancer (CRC) remain controversial. This study aimed to explore how preoperative anemia affects the long-term survival of patients undergoing colorectal cancer surgery.

**Methods:**

This was a retrospective cohort study in which adult patients underwent surgical resection for colorectal cancer between January 1, 2008, and December 31, 2014, at a large tertiary cancer center. A total of 7436 patients were enrolled in this study. Anemia was defined according to the diagnostic criteria of China (hemoglobin level < 110 g/L for women and < 120 g/L for men). The median follow-up time was 120.5 months (10.0 years). Inverse probability of treatment weighting (IPTW) using the propensity score was used to reduce selection bias. Overall survival (OS) and disease-free survival (DFS) were compared between patients with and without preoperative anemia using the Kaplan–Meier estimator and the weighted log-rank test based on IPTW. Univariate and multivariate Cox proportional hazards models were used to assess factors associated with OS and DFS. Multivariable Cox regression was also used to assess red blood cell (RBC) transfusion associations between preoperative anemia and outcomes.

**Results:**

After IPTW adjustment, clinical profiles were similar, except that tumor location and TNM stage remained imbalanced between the preoperative anemia and preoperative non-anemia groups (*p* < 0.001). IPTW analysis showed that the 5-year OS rate (71.3 vs. 78.6%, *p* < 0.001) and the 5-year DFS rate (63.9 vs. 70.9%, *p* < 0.001) were significantly lower in the preoperative anemia group. Multivariate analysis showed that preoperative anemia was associated with poorer OS and DFS, while RBC transfusion may improve OS (hazard ratio [HR] 0.54, *p* = 0.054) and DFS (HR 0.50, *p* = 0.020) in CRC patients with preoperative anemia.

**Conclusions:**

Preoperative anemia is an independent risk factor for survival in patients undergoing colorectal surgery. Strategies to reduce preoperative anemia in patients with CRC should be considered.

**Supplementary Information:**

The online version contains supplementary material available at 10.1186/s12957-023-03005-w.

## Background

Colorectal cancer (CRC) is the third most common cancer in men and the second most common cancer in women [1, 2]. Surgical resection of the primary tumor remains one of the major curative treatment options available to patients with CRC. Nevertheless, patients with CRC have a high prevalence of anemia [3], which likely results from iron deficiency, systemic inflammation, adjuvant chemotherapy, spontaneous tumor bleeding, or surgical blood loss, and it is reported in up to 40% of patients, half of whom have moderate to severe anemia [4, 5]. Besides being an important marker of a more advanced tumor stage and treatment intensity, anemia in cancer is also a well-established risk factor for infection, impaired physical function, and inferior survival [6]. Furthermore, evidence that preoperative anemia, which is common in patients undergoing CRC surgery, is associated with long-term adverse outcomes in patients with CRC and should be treated in a timely manner remains controversial [7–10].

The association of preoperative anemia with the overall survival (OS) and disease-free survival (DFS) of patients with CRC is still disputed. In clinical practice, the influence of preoperative anemia on outcomes is often confounded by perioperative blood transfusion. Perioperative anemia is a major risk factor for blood transfusion, which leads to immunosuppressive effects in surgical patients. In addition to increasing in organ dysfunction and infection risk [11], studies have documented that allogeneic blood transfusion for the treatment of anemia in patients with cancer may also promote cancer progression and recurrence [12]. However, the theoretical disadvantages of perioperative anemia and perioperative blood transfusion did not always translate into worse oncological outcomes in previous studies [9, 13]. Even though perioperative anemia itself poses a significant risk for postoperative complications in cancer patients, its effects on OS and DFS of the patients undergoing surgery for cancers are inconclusive, since previous studies had a small sample size and heterogeneity in study design [3, 9]. To further clarify the risk factors associated with anemia and the impacts of preoperative anemia on long-term survival outcomes in patients with CRC, we performed this retrospective study in a single cancer center with a big database of patients with CRC. This study aimed to investigate whether preoperative anemia was associated with OS and DFS after surgery in patients with CRC.

## Methods

This retrospective study was approved by the Ethics Committee of the Fudan University Shanghai Cancer Center (FUSCC), China. The requirement for written informed consent was waived by the Ethics Committee of FUSCC, and all datasets were anonymized and de-identified before analysis. All clinical data were collected in accordance with the principles of the Declaration of Helsinki. All work complied with the STROBE (Strengthening the Reporting of Observational Studies in Epidemiology) guidelines [14].

### Study design and patients

From January 1, 2008, to December 31, 2014, at FUSCC, 7436 consecutive patients who underwent elective resections for CRC were retrospectively enrolled in this cohort study and followed up until death or December 31, 2020. Eligibility criteria included elective curative surgery, histologically confirmed CRC, and age > 20 years. Patients with recurrent cancer, organ metastases, or palliative non-resectable surgery were excluded. Patients with incomplete medical records were also excluded from this study.

Data were extracted from the FUSCC Clinical Information System database. The medical information of each patient was reviewed and recorded, including demographic data (sex and age), primary diagnosis, medical history including preoperative chemotherapy, pathological details (tumor location, differentiation, type, TNM stage, and histological diagnosis), preoperative and postoperative hemoglobin (Hb) levels, perioperative red blood cell transfusion volumes, and postoperative outcomes. Patients were followed up every 3 months for the first 2 years after surgery, every 6 months thereafter for 3 years and then every 1 year after 5 years. Medical history, physical examination, and serum carcinoembryonic antigen (CEA) levels were examined at each follow-up visit. Abdominopelvic and chest computed tomography (CT) scans were performed every 6 months, while colonoscopy was performed annually.

### Outcomes and anemia definitions

The primary outcomes were overall survival (OS) and disease-free survival (DFS) after surgery in CRC patients. The second outcomes were postoperative recovery parameters, including the length of postoperative hospitalization, incidence of postoperative anemia, readmission within 30 days, and mortality. OS was defined as the length of time from the date of definite diagnosis to the date of death or to December 31, 2020. DFS was defined as the length of time from the date of definite diagnosis to the date of the first evidence of tumor recurrence or the last follow-up date. Tumor recurrence was defined by imaging studies and colonoscopic examination and confirmed by colonoscopic or percutaneous biopsy [15]. Perioperative red blood cell (RBC) transfusion was defined as intraoperative and postoperative in-hospital transfusion. On the other hand, anemia in this study was defined as an Hb level < 110 g/L for women and < 120 g/L for men according to the diagnostic criteria in China [16], but not from the World Health Organization (WHO) [17].

### Statistical analysis

Continuous variables were described as the mean ± SD or median (interquartile range) and compared using the *t* test or, in cases of non-normality, the Mann–Whitney test. Categorical variables are presented as frequencies and percentages and were compared using the *χ*^2^ or Fisher’s exact test.

To adjust for selection bias and potential confounding factors between patient groups in comparisons of outcomes, stabilized inverse probability of treatment weighting (IPTW) based on propensity scores was performed to control for differences in baseline characteristics between preoperative anemic and non-anemic patients. A logistic regression model was used to calculate propensity scores including the covariates: sex, age, preoperative chemotherapy, preoperative CEA, surgical approach, tumor location, tumor type, tumor differentiation, vascular cancer embolus, nerve invasion, surgical margin, TNM stage, perioperative allogeneic RBC transfusion, and amount of blood loss. Standardized mean differences (SMD) were used to measure the balance of individual covariates before and after IPTW. Differences were considered statistically significant at SMD > 10%.

Survival rates were computed using the Kaplan–Meier method. IPTW-adjusted Kaplan–Meier survival curves and the weighted log-rank test were generated by comparing the preoperative anemia groups. The effects of anemia and other potential prognostic factors presented as IPTW-adjusted hazard ratios (HRs) and 95% confidence intervals (CIs) were estimated using the weighted Cox proportional hazards model. Variables with a *p* value less than 0.05 in the univariate analysis and those variables still unbalanced between groups after IPTW were included in the multivariable regression analysis to mitigate the analytic bias. Hypothesis testing was performed at a two-sided 5% significance level. Statistical analyses were performed using R version 3.4.3 (R Foundation for Statistical Computing).

## Results

A total of 7436 patients underwent elective curative surgery for CRC and were enrolled in this study. Surgery and lymph node harvest were performed according to oncological standards, yielding resection margins of at least 10 cm for colon cancer and total mesorectal excision for rectal cancer. Of the 7436 study patients, 1747 (23.5%) presented with preoperative anemia. The median follow-up time of the 7436 patients was 120.5 months (interquartile range 114.0–127.0 months). Of the patients in the anemia and non-anemia groups, 130 (7.4%) and 42 (0.7%) received perioperative allogenic RBC transfusion (*p* < 0.001), respectively (Table 1). Compared with patients in the non-anemia group, anemic patients were older (60.2 ± 13.5 years vs. 58.3 ± 11.6 years, *p* < 0.001), received preoperative chemotherapy more frequently (10.2 vs. 7.5%, *p* < 0.001), had higher preoperative serum CEA levels (3.7 ng/mL vs. 2.8 ng/mL, *p* < 0.001), had worse tumor differentiation (23.0 vs. 18.8%, *p* < 0.001), and had a more advanced TNM stage (stages III–IV 51.9 vs. 47.2%, *p* < 0.001).

**Table 1:** Patient characteristics of the preoperative anemia group and preoperative non-anemia group before and after IPTW

Variables	Entire study population				Weighted covariates			
	Anemia (*n* = 1747)	Non-anemia (*n* = 5689)	*P* value	Standardized difference (%)	Anemia (*n* = 1695.7)	Non-anemia (*n* = 5810.6)	*P* value	Standardized difference (%)
Gender			< 0.001	14.2			0.492	2.1
Female	803 (46.0)	2216 (39.0)			714.1 (42.1)	2386.7 (41.1)		
Male	944 (54.0)	3473 (61.0)			918.5 (57.9)	3424.0 (58.9)		
Age, years	60.2 ± 13.5	58.3 ± 11.6	< 0.001	15.0	58.2 ± 13.9	58.7 ± 11.7	0.241	3.9
Preoperative chemotherapy			< 0.001	9.7			0.322	2.8
No	1568 (89.8)	5263 (92.5)			1544.8 (91.1)	5338.7 (91.9)		
Yes	179 (10.2)	426 (7.5)			150.9 (8.9)	471.9 (8.1)		
Preoperative CEA, median (IQR), ng/mL	3.7 (1.9, 10.6)	2.8 (1.7, 6.0)	< 0.001	11.1	3.4 (1.6, 9.9)	2.7 (1.6, 5.9)	0.562	3.4
Surgical approach			0.005	8.1			0.892	0.4
Laparotomy	1610 (93.8)	5215 (91.7)			1563.4 (92.2)	5364.0 (92.3)		
Laparoscopy	109 (6.2)	474 (8.3)			132.3 (7.8)	446.6 (7.7)		
Tumor location			< 0.001	64.1			< 0.001	34.9
Rectum	609 (34.9)	3423 (60.2)			805.3 (47.5)	3179.8 (54.7)		
Left-side colon	326 (18.7)	1197 (21.0)			330.5 (19.5)	1193.1 (20.5)		
Right-side colon	767 (43.9)	1010 (17.8)			543.9 (32.1)	1185.8 (20.4)		
Entire colon	7 (0.4)	5 (0.1)			5.4 (0.3)	6.5 (0.1)		
Cannot distinguish left or right-side colon	38 (2.2)	54 (0.9)			10.6 (0.6)	245.3 (4.2)		
Tumor type			< 0.001	16.6			0.026	8.6
Adenocarcinoma	1441 (82.5)	5008 (88.0)			1457.8 (86.0)	5015.6 (86.3)		
Mucoid adenocarcinoma	281 (16.1)	595 (10.5)			220.9 (13.0)	681.9 (11.7)		
Signet-ring cell carcinoma	25 (1.4)	86 (1.5)			17.0 (1.0)	113.2 (1.9)		
Tumor differentiation			< 0.001	13.4			0.053	9.4
Poor	402 (23.0)	1070 (18.8)			339.9 (20.0)	1181.8 (20.3)		
Moderate	1148 (65.7)	3900 (68.6)			1153.3 (68.0)	3875.3 (66.7)		
Well	22 (1.3)	143 (2.5)			21.9 (1.3)	149.0 (2.6)		
Unknown	175 (10.0)	576 (10.1)			180.6 (10.7)	604.5 (10.4)		
Vascular cancer embolus			0.214	3.5			0.947	0.2
Negative	1356 (77.6)	4497 (79.0)			1323.1 (78.0)	4528.9 (77.9)		
Positive	391 (22.4)	1192 (21.0)			372.6 (22.0)	1281.7 (22.1)		
Nerve invasion			0.257	3.2			0.585	1.8
Negative	1450 (83.0)	4652 (81.8)			1373.8 (81.0)	4747.8 (81.7)		
Positive	397 (17.0)	1037 (18.2)			321.9 (19.0)	1062.8 (18.3)		
Surgical margin			0.314	3.0			0.579	1.9
Negative	1718 (98.3)	5615 (98.7)			1671.1 (98.6)	5712.4 (98.3)		
Positive	29 (1.7)	74 (1.3)			24.6 (1.4)	98.2 (1.7)		
TNM stage			< 0.001	35.4			< 0.001	25.8
0–I	171 (9.8)	1232 (21.7)			208.5 (12.3)	1196.4 (20.6)		
II	629 (36.0)	1602 (28.2)			592.4 (34.9)	1641.7 (28.3)		
III	779 (44.6)	2413 (42.4)			757.7 (44.7)	2462.7 (42.4)		
IV	127 (7.3)	273 (4.8)			106.3 (6.3)	323.7 (5.6)		
Unknown	41 (2.3)	168 (3.0)			30.9 (1.8)	186.2 (3.2)		
Perioperative allogenic RBC transfusion^a^			< 0.001	34.3			0.887	0.6
No	1617 (92.6)	5647 (99.3)			1656.2 (97.7)	5670.4 (97.6)		
Yes	130 (7.4)	42 (0.7)			39.5 (2.3)	140.2 (2.4)		
Amount of blood loss			0.835	1.0			0.160	6.3
< 400 ml	1731 (99.1)	5642 (99.2)			1669.2 (98.4)	5759.9 (99.1)		
≥ 400 ml	16 (0.9)	47 (0.8)			26.4 (1.6)	50.7 (0.9)		

The values were expressed as mean ± SD or median (interquartile range, IQR) or number (%)

*Abbreviations: CEA* Carcinoembryonic antigen, *IPTW* Inverse probability of treatment weights, *RBC* Red blood cell, *TNM* Tumor nodes metastasis

^a^Use of perioperative allogenic RBC transfusion was defined as a receipt at least 1 unit of packed RBCs during a patient’s in-hospital admission (from the time of primary surgery to hospital discharge)

**Fig. 1:**
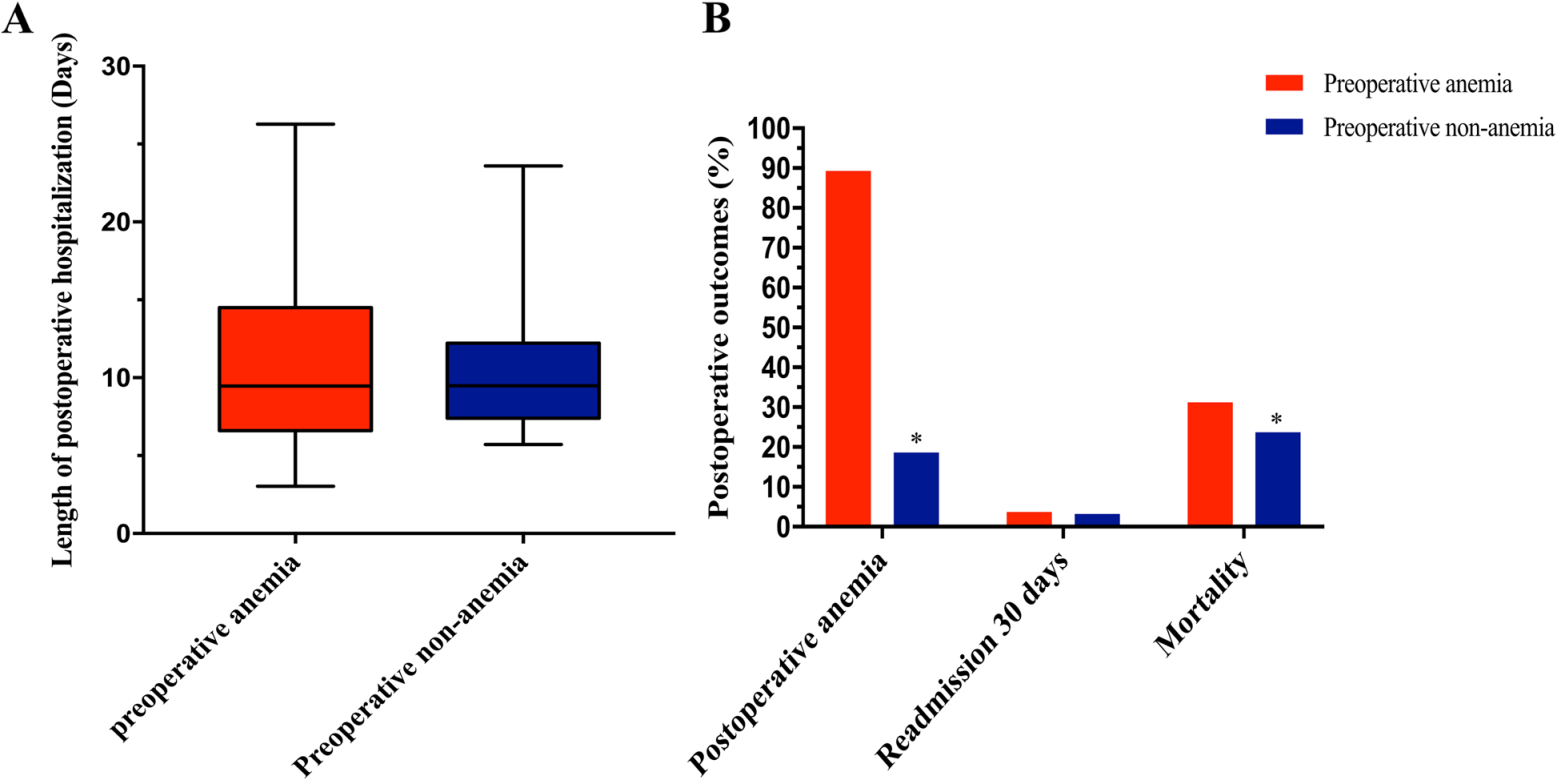
Postoperative recovery outcomes after weighting. **A** The length of postoperative hospitalization had no significant differences between preoperative anemia and non-anemia groups (median time 9.5 vs. 9.5 days, *p* = 0.275). **B** The patients in the preoperative anemia group more likely had postoperative anemia (89.3 vs. 18.6%, *p* < 0.001) and higher mortality (31.2 vs. 23.7%, *p* < 0.001), but similar on 30-day readmission rates (3.7 vs. 3.2%, *p* = 0.262). *p < 0.001

IPTW was used to reduce the imbalance in baseline characteristics between the two groups. After weighting, 1695.7 (22.6%) of patients presented with preoperative anemia (Table 1). The groups were similar after IPTW, except that tumor type (*p* = 0.026), tumor location (*p* < 0.001), and TNM stage (*p* < 0.001) remained unbalanced between the two groups. The results of the univariate and multivariate analyses of OS and DFS are shown in Table 2. On univariate analysis of survival, preoperative anemia was associated with a strong trend toward worse OS (HR 1.54; 95% CI 1.39–1.70; *p* < 0.001) as well as on multivariable analysis (HR 1.36; 95% CI 1.20–1.55; *p* < 0.001). As for DFS, the presence of preoperative anemia was a significant factor in the univariate analysis (HR 1.33; 95% CI 1.20–1.47; *p* < 0.001), as well as in the multivariate analysis (HR 1.28; 95% CI 1.15–1.43; *p* < 0.001). Additionally, patients in the preoperative anemia group were more likely to have postoperative anemia (89.3 vs. 18.6%, *p* < 0.001) and higher mortality (31.2 vs. 23.7%, *p* < 0.001, Fig. 1B). The 30-day readmission rate (3.7 vs. 3.2%, *p* = 0.262) and length of postoperative hospitalization (median time 9.5 days vs. 9.5 days, *p* = 0.275) were not significantly different between the preoperative anemia and non-anemia groups (Fig. 1).

**Table 2:** Univariate analysis and multivariate analysis of the overall survival and disease-free survival after IPTW

Variables	Univariate analysis		Multivariate analysis	
	HR (95%CI)	*P* value	Adjusted HR (95%CI)	*P* value
**Overall survival**				
Preoperative anemia		< 0.001		< 0.001
No	1 (reference)		1 (reference)	
Yes	1.54 (1.39, 1.70)		1.36 (1.20, 1.55)	
Tumor location				
Rectum	1 (reference)		1 (reference)	
Left-side colon	0.92 (0.81, 1.04)	0.182	0.79 (0.69, 0.90)	< 0.001
Right-side colon	1.09 (0.97, 1.21)	0.141	0.98 (0.83, 1.16)	0.790
Entire colon	0.39 (0.06, 2.80)	0.352	0.19 (0.02, 1.65)	0.132
Cannot distinguish left or right-side colon	1.00 (0.66, 1.53)	0.994	0.76 (0.42, 1.36)	0.355
Tumor type				
Adenocarcinoma	1 (reference)		1 (reference)	
Mucoid adenocarcinoma	1.25 (1.09, 1.42)	0.001	1.17 (0.95, 1.45)	0.144
Signet-ring cell carcinoma	2.98 (2.27, 3.91)	< 0.001	2.69 (1.64, 4.42)	< 0.001
TNM stage				
0–I	1 (reference)		1 (reference)	
II	1.44 (1.20, 1.73)	< 0.001	1.38 (1.13, 1.68)	0.002
III	3.17 (2.68, 3.74)	< 0.001	3.10 (2.60, 3.70)	< 0.001
IV	13.75 (11.33, 16.70)	< 0.001	12.96 (10.28, 16.35)	< 0.001
Unknown	0.89 (0.56, 1.41)	0.609	0.90 (0.56, 1.46)	0.677
**Disease-free survival**				
Preoperative anemia		< 0.001		< 0.001
No	1 (reference)		1 (reference)	
Yes	1.33 (1.20, 1.47)		1.28 (1.15, 1.43)	
Tumor location				
Rectum	1 (reference)		1 (reference)	
Left-side colon	0.93 (0.84, 1.04)	0.209	0.83 (0.74, 0.93)	0.002
Right-side colon	1.11 (0.97, 1.26)	0.138	0.99 (0.86, 1.14)	0.901
Entire colon	0.86 (0.26, 2.81)	0.802	0.55 (0.14, 2.12)	0.385
Cannot distinguish left or right-side colon	0.96 (0.60, 1.53)	0.857	0.85 (0.52, 1.38)	0.506
Tumor type				
Adenocarcinoma	1 (reference)		1 (reference)	
Mucoid adenocarcinoma	1.29 (1.09, 1.54)	0.004	1.17 (0.98, 1.39)	0.090
Signet-ring cell carcinoma	2.65 (1.68, 4.18)	< 0.001	2.00 (1.27, 3.14)	0.003
TNM stage				
0–I	1 (reference)		1 (reference)	
II	1.40 (1.20, 1.65)	< 0.001	1.36 (1.15, 1.60)	< 0.001
III	2.87 (2.48, 3.32)	< 0.001	2.75 (2.38, 3.17)	< 0.001
IV	9.36 (7.58, 11.55)	< 0.001	9.26 (7.49, 11.44)	< 0.001
Unknown	0.95 (0.65, 1.39)	0.800	0.95 (0.65, 1.39)	0.789

*Abbreviations: CI* Confidence interval, *HR* Hazard ratio, *IPTW* Inverse probability of treatment weights, *TNM* Tumor nodes metastasis

**Fig. 2:**
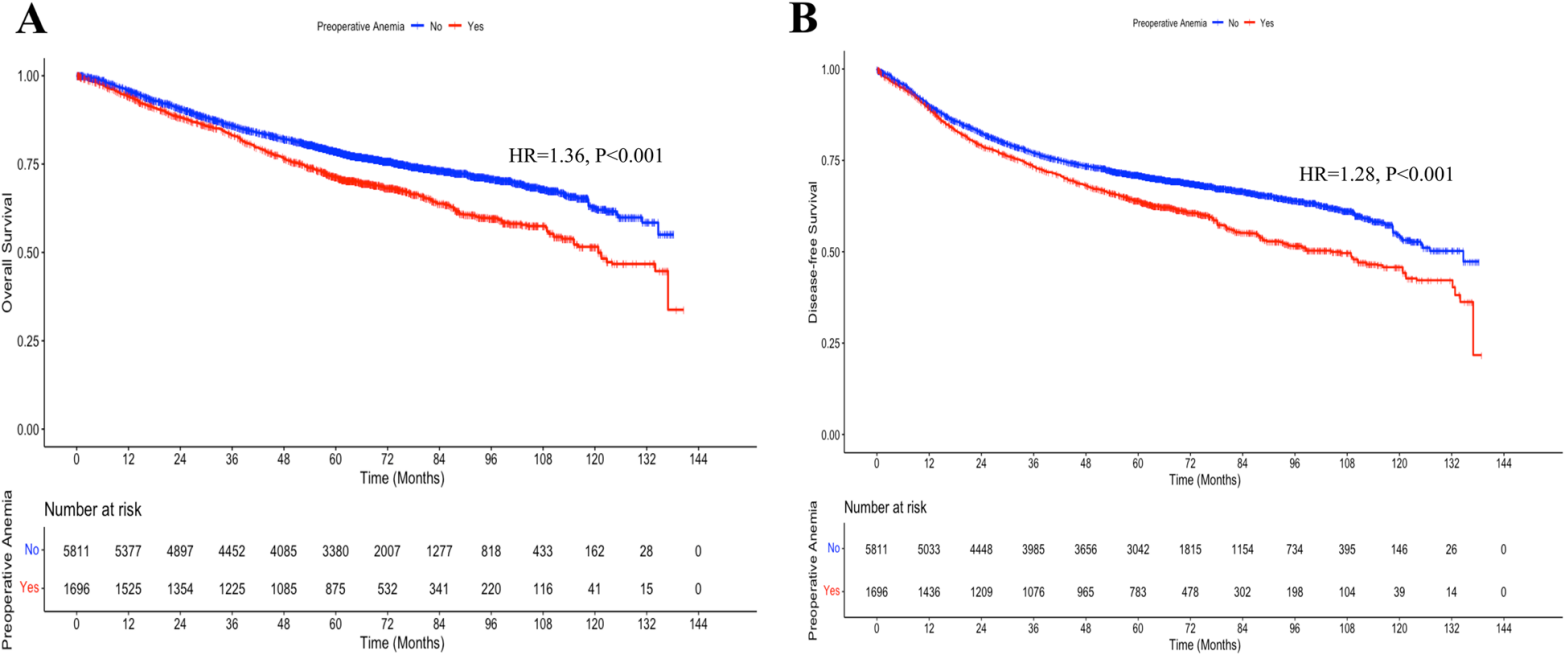
Overall survival (OS) and disease-free survival (DFS) after weighting by the Kaplan–Meier method. **A** The 5-year OS rate was significantly worse in the preoperative anemia group than in the non-anemia group (71.3% vs. 78.6%, *p* < 0.001). **B** The preoperative anemia was also associated with a significantly worse 5-year DFS (63.9% vs. 70.9%, *p* < 0.001)

**Fig. 3:**
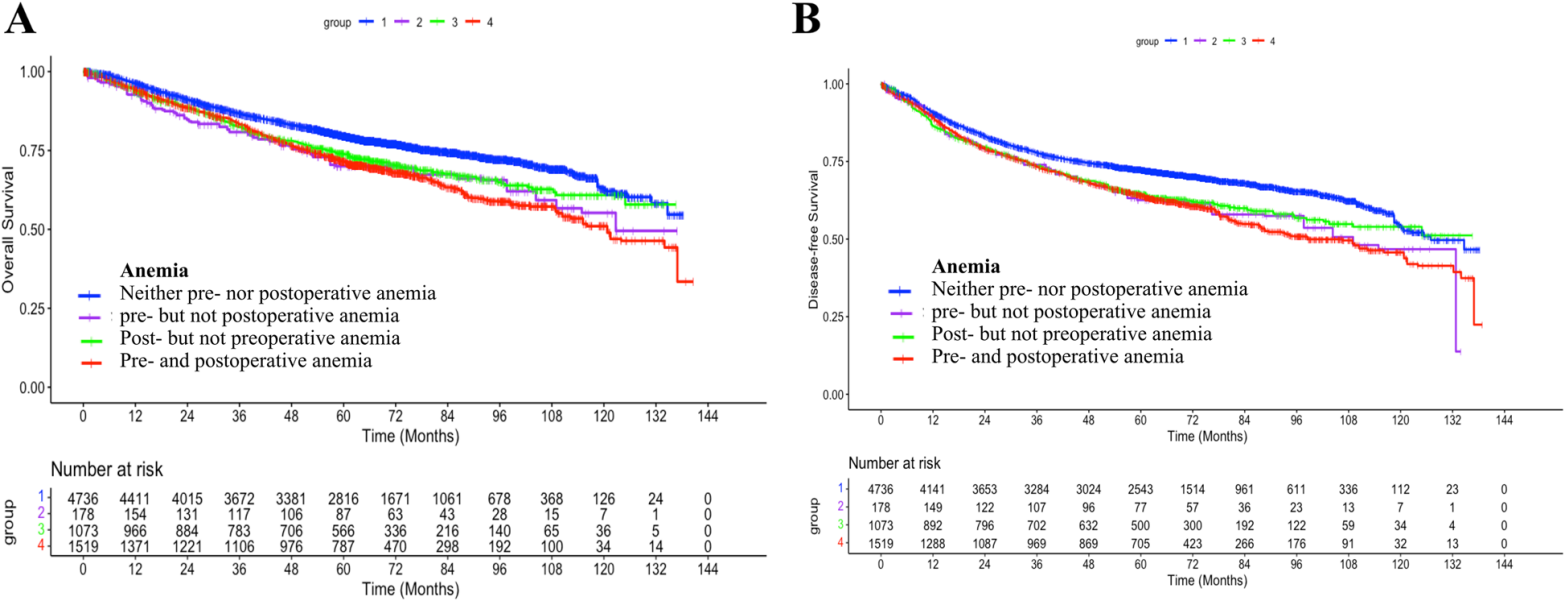
Overall survival (OS) and disease-free survival (DFS) after weighting by the Kaplan–Meier method when pre- and post-operative anemia were both considered. **A** When preoperative and postoperative anemia were both considered, the 5-year OS rates were 71.5%, 70.0%, 73.9%, and 79.6% in the combined preoperative and postoperative anemia, preoperative but not postoperative anemia, postoperative but not preoperative anemia and non-anemia groups, respectively (*p* < 0.001). **B** When preoperative and postoperative anemia were both considered, the 5-year DFS rates were 64.1%, 62.6%, 64.6%, and 72.3%, respectively, in these four groups (*p* < 0.001)

The Kaplan–Meier curve showed that the 5-year OS rate was significantly lower in the preoperative anemia group than in the non-anemia group (71.3% vs. 78.6%, *p* < 0.001, Fig. 2A). When pre- and post-operative anemia were both considered, the 5-year OS rates were 71.5% (combined pre- and post-operative anemia), 70.0% (preoperative anemia only), 73.9% (postoperative anemia only), and 79.6% (non-anemia) (*p* < 0.001, Fig. 3A). Overall, the 5-year DFS rate was lower in the preoperative anemia group than in the preoperative non-anemia group (63.9% vs. 70.9%, *p* < 0.001, Fig. 2B). Similarly, the 5-year DFS rates were 64.1% (combined pre- and post-operative anemia), 62.6% (preoperative anemia only), 64.6% (postoperative anemia only), and 72.3% (non-anemia) (*p* < 0.001, Fig. 3B) when pre- and post-operative anemia were both considered. Then, we compared the survival prognosis of preoperative anemia vs. non-anemia in advanced cancer patients. After being screened (TNM stages III–IV and tumor differentiation: poor), there were 971 patients (preoperative anemia group: 253 vs. preoperative non-anemia group, 718), with prognosis at high risk and 6465 patients (preoperative anemia group, 1494 vs. preoperative non-anemia group, 4971) with low-moderate risk. The analysis showed that preoperative anemia was an independent risk factor of worse survival outcomes for those patients with low-moderate risk but not for those with high risk. The results of their OS and DFS after IPTW are listed in Supplementary Table 1.

**Fig. 4:**
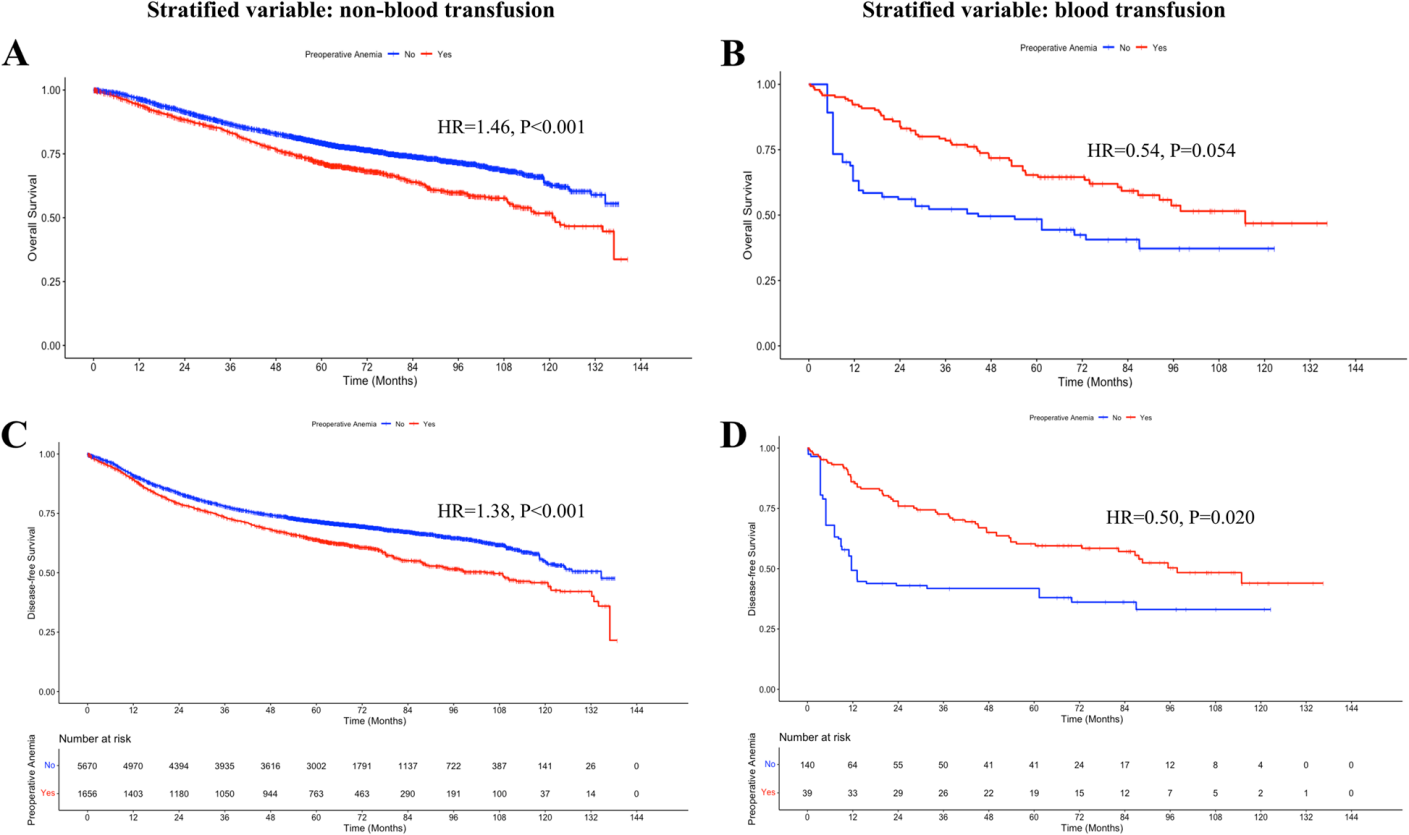
The interaction between preoperative anemia and perioperative blood transfusion after weighting. **A**,** C** When non-transfusion was the stratified variable, the preoperative anemia group has poorer overall survival (OS) and disease-free survival (DFS) than the preoperative non-anemia group; **B**,** D** When blood transfusion was the stratified variable, the preoperative anemia group has a better outcome in most follow-up, especially for DFS

We also assessed perioperative RBC transfusion associations between preoperative anemia and outcomes using interaction terms. The results showed that the OS and DFS rates were lower in the preoperative anemia group than in the preoperative non-anemia group (OS, HR; 1.46, *p* < 0.001; DFS, HR 1.38, *p* < 0.001, Fig. 4A, C) when the non-RBC transfusion was stratified, further confirming that preoperative anemia is an independent risk factor for poor long-term prognosis. In contrast, transfused patients without preoperative anemia seemed to have a poorer OS than transfused patients with preoperative anemia during most follow-ups when RBC transfusion was stratified (HR 0.54, *p* = 0.054; Fig. 4B). Most importantly, these findings suggest that RBC transfusion could improve DFS in patients with CRC with preoperative anemia (HR 0.50, *p* = 0.0.020, Fig. 4D).

## Discussion

The overall prevalence of preoperative anemia was 23.5% in our center, lower than that (40%) reported in other studies [5, 18], which can be explained by the definition of anemia adopted in this study. Our major findings demonstrate that preoperative anemia alone and combined pre- and post-operative anemia, rather than postoperative anemia alone, are at an elevated risk for worse OS and DFS. In contrast, patients without anemia had the best OS and DFS among patients undergoing curative surgery for colorectal cancer. These results suggest that preoperative anemia is an independent risk factor for long-term adverse outcomes in this population. Furthermore, perioperative allogeneic RBC transfusion may be beneficial to the DFS of patients with preoperative anemia. The preoperative optimization of the Hb level with the implementation of a preoperative anemia management protocol in anemic colorectal cancer patients may be favorable for the reduction in perioperative transfusion and postoperative morbidity, which is consistent with the results of previous studies [19, 20].

The etiology of anemia in patients with cancer is multifactorial [21]. Poor intake or malabsorption leads to nutritional deficiencies (e.g., iron, vitamin B12, folate, and proteins). Several medications (e.g., chemotherapeutics and metformin) also contribute to reduced RBC production. Iron deficiency is the most common cause of cancerous anemia due to nutritional deficiency and blood loss characterized by low iron stores. Additionally, these populations often have other chronic diseases that disrupt the normal pathways for iron transport and metabolism [22]. Recently, it was reported that preoperative intravenous iron supplements neither elevated Hb concentrations at the time of surgery nor reduced the likelihood of receiving an RBC transfusion in CRC patients with iron deficiency anemia [23]. The overall postoperative complication rate was higher in patients receiving intravenous iron treatment. Although the role of perioperative allogeneic RBC transfusion in the survival outcome of patients with CRC remains controversial, RBC transfusion is still the only way to correct anemia quickly and effectively. Our findings indicated that preoperative anemia was an independent risk factor associated with worse survival in CRC patients; however, for those patients with advanced cancer, the cancer-associated systemic failures rather than preoperative anemia alone might be the main contributor of poor survival [15]. As preoperative anemia is a modifiable risk factor, the effect of preoperative RBC transfusion on the survival of anemic patients with CRC should be further confirmed.

At present, the main treatment of anemia consists of RBC transfusions and several alternatives to blood transfusions, such as preoperative iron and vitamins or folate supplementation, autologous blood donation, or administration of recombinant human erythropoietin [24]. However, little effort has been made to correct preoperative anemia before surgery because of the limited preoperative preparation time and shortage of blood products. Yet, virtually anemic patients are at high risk of receiving perioperative allogeneic RBC transfusion [25]. Currently, no specific recommendations or guidelines have been proposed for the evaluation or treatment of anemia prior to surgery. Our results indicated that only 2.3% of patients were transfused with packed RBC, and most of them were in the preoperative anemia group. Whether preoperative anemia or blood transfusion is the culprit for worse outcomes in patients with CRC is not clear [26, 27]. A transfusion reduction initiative suggested that reducing blood transfusion did not prolong DFS in CRC patients [28]. The PREVENTT trial, a large multicenter clinical study, suggested that preoperative intravenous iron supplements in patients with anemia before major open elective abdominal surgery cannot reduce the need for blood transfusion or mortality in the perioperative period [29, 30]. Specially, a dose of 1000–2000 mg preoperative intravenous iron therapy was found not to have an impact on a long-term overall and disease-free survival in anemic colorectal cancer patients [31]. The systematic review also indicated that preoperative intravenous iron therapy for anemic patients undergoing abdominal surgery could substantially increase Hb levels but minimally reduce the incidence of allogeneic blood transfusions or improve clinical outcomes [32]. This indirectly indicated that correcting anemia prior to surgery by RBC transfusion was feasible and practical. However, several studies showed that perioperative blood transfusion was associated with worse survival in patients undergoing surgery for colorectal cancer [33, 34]. Nevertheless, a study examining the interaction between preoperative anemia and perioperative transfusions with postoperative short-term mortality in patients undergoing gastrectomy for cancer revealed that perioperative transfusions appeared to be beneficial only for preoperative hematocrit values < 29% [35]. Our study suggests that this may also be true for colorectal cancer, at least within the first year after surgery. However, this benefit became less significant during longer follow-up periods, similar to findings in studies on non-small cell lung cancer [36]. Therefore, the differential effects of RBC transfusion in anemic and non-anemic CRC patients should be evaluated and individualized RBC transfusion strategy for patients with CRC should be provided accordingly.

Postoperative anemia, commonly resulting from perioperative blood loss or preoperative anemia, is reported to be strongly associated with postoperative ischemic events in patients undergoing major general and vascular surgery [37], which may pose a risk of a short-term postoperative mortality. Our results showed that due to better preoperative health conditions, the patients with postoperative anemia alone had a better outcome than those with preoperative anemia. When combined pre- and post-operative anemia were both considered, the 5-year OS and DFS rates were slightly worse than those of preoperative anemia alone, suggesting the risk of death was slightly amplified by postoperative anemia in patients with preoperative anemia.

The advantage of this study is the relatively large sample size, which has been the largest so far, to the best of our knowledge, for assessing the impact of preoperative anemia on long-term survival outcomes and providing data supporting the benefit of RBC transfusion on DFS in patients undergoing surgery for CRC. There are also some inherent disadvantages in the retrospective cohort and observational studies. First, it is inevitable for an observational database to include the inability to establish causality and missing data, particularly baseline laboratory values. Several variables including Charlson score, nutritional status, baseline frailty, ASA classification, duration of surgery, and Clavien-Dindo grade are not available because they failed to be extracted from the FUSCC clinical information system database. As the confounders for outcomes in anemic patients, these missing data may weaken the strength of our conclusions. Second, Hb levels remain the most widely accepted laboratory parameter for the diagnosis of anemia, even though they are not true markers of RBC mass or tissue oxygen delivery [38] and are influenced by physiological changes associated with aging, such as declining production and shortening the lifespan of RBCs [39]. The WHO definition of anemia (< 13 g/dL in men and < 12 g/dL in women), which is different with that in China (< 12 g/dL in men and < 11 g/dL in women) adopted in this study, is derived from the distribution of Hb values in epidemiologic investigations and not by the clinical and physiological impact of those values [40, 41], thereby it may influence the results of a long-term survival following CRC surgery. Finally, although IPTW using the propensity score has highly mitigated the inter-group imbalances, some clinical baseline variables still did not match well; nevertheless, multivariable Cox regression could ensure that the final results are reliable.

## Conclusions

In summary, our findings show that preoperative anemia is common in CRC patients and is an independent risk factor significantly associated with worse outcomes in patients undergoing surgery for CRC, especially for those with a combination of preoperative and postoperative anemia. As anemia is also prevalent in other malignancies, efforts to optimize preoperative Hb levels should be made, as it is a potentially modifiable risk factor for long-term survival in the cancer population.

## Supplementary Information

**Additional file 1:** **Supplementary Table 1.** The overall survival and disease-free survival after IPTW in patients with prognosis at high risk and those with low-moderate risk.

## Data Availability

All data generated or analyzed during this study are available from the corresponding author on reasonable request.

## Abbreviations

CRC: Colorectal cancer
CT: Computed tomography
DFS: Disease-free survival
FUSCC: Fudan University Shanghai Cancer Center
Hb: Hemoglobin
IPTW: Inverse probability of treatment weighting
OS: Overall survival
PRC: People’s Republic of China
RBC: Red blood cell
STROBE: Strengthening the Reporting of Observational Studies in Epidemiology
TNM: Tumor nodes metastasis.
WHO: World Health Organization
