# Supplementary material for: Preoperative anemia and long-term survival in patients undergoing colorectal cancer surgery: a retrospective cohort study
Source: World J Surg Oncol. 2023 Apr 4;21:122. doi: 10.1186/s12957-023-03005-w (PMC10071685; doi:10.1186/s12957-023-03005-w)
Supplement: Supplementary file 1 — Additional file 1: Supplementary Table 1. The overall survival and disease-free survival after IPTW in patients with prognosis at high risk and those with low-moderate risk. [file 12957_2023_3005_MOESM1_ESM.docx]

**Supplementary Table 1** The overall survival and disease-free survival after IPTW in patients with prognosis at high risk and those with low-moderate risk

| **Variables** | | **HR (95% CI)** | **P Value** | |
| --- | --- | --- | --- | --- |
| **Prognosis at high risk^*^ (preoperative anemia group: 253 vs. non-anemia group: 718)** | | | | |
| **Overall Survival** | |  |  | |
| Preoperative anemia | |  | 0.312 | |
| No | | 1(reference) |  | |
| Yes | | 0.88 (0.68, 1.13) |  | |
| **Disease-free Survival** | |  |  | |
| Preoperative anemia | |  | 0.170 | |
| No | | 1(reference) |  | |
| Yes | | 0.85 (0.68, 1.07) |  | |
|  | |  |  | |
| **Prognosis at low-moderate risk^#^ (preoperative anemia group:1494 vs. non-anemia group: 4971)** | | | | |
| **Overall Survival** |  | | |  |
| Preoperative anemia |  | | | <0.001 |
| No | 1(reference) | | |  |
| Yes | 1.62(1.43, 1.85) | | |  |
| **Disease-free Survival** |  | | |  |
| Preoperative anemia |  | | | <0.001 |
| No | 1(reference) | | |  |
| Yes | 1.49(1.33, 1.66) | | |  |

Abbreviations: CI, confidence interval; HR, hazard ratio.

*: high risk: III-IV TNM staging and poor tumor differentiation;

#: low-moderate risk: III-IV TNM staging only; or poor tumor differentiation only; or none.
